# Combined use of Panax notoginseng and leech provides new insights into renal fibrosis: Restoration of mitochondrial kinetic imbalance

**DOI:** 10.1371/journal.pone.0303906

**Published:** 2024-05-29

**Authors:** Xin Chen, Jingwei Deng, Ling Zuo, Hongyu Luo, Munan Wang, Peng Deng, Kang Yang, Qian Yang, Xuekuan Huang

**Affiliations:** 1 College of Traditional Chinese Medicine, Chongqing Medical University, Chongqing, China; 2 Chongqing Key Laboratory of Traditional Chinese Medicine for Prevention and Cure of Metabolic Diseases, Chongqing, China; Brigham and Women’s Hospital, UNITED STATES

## Abstract

In this study, we aimed to investigate the protective effects of Panax notoginseng and leech (PL) on renal fibrosis and explore the mechanisms underlying their actions. For this study, we created an adenine-induced renal fibrosis model in SD rats to investigate the protective effect of PL on renal fibrosis and explore its underlying mechanism. Initially, we assessed the renal function in RF rats and found that Scr, BUN, and urine protein content decreased after PL treatment, indicating the protective effect of PL on renal function. Histological analysis using HE and Masson staining revealed that PL reduced inflammatory cell infiltration and decreased collagen fiber deposition in renal tissue. Subsequently, we analyzed the levels of α-SMA, Col-IV, and FN, which are the main components of the extracellular matrix (ECM), using IHC, RT-qPCR, and WB. The results demonstrated that PL was effective in reducing the accumulation of ECM, with PL1-2 showing the highest effectiveness. To further understand the underlying mechanisms, we conducted UPLC-MS/MS analysis on the incoming components of the PL1-2 group. The results revealed several associations between the differential components and antioxidant and mitochondrial functions. This was further confirmed by enzyme-linked immunosorbent assay and biochemical indexes, which showed that PL1-2 ameliorated oxidative stress by reducing ROS and MDA production and increasing GSH and SOD levels. Additionally, transmission electron microscopy results indicated that PL1-2 promoted partial recovery of mitochondrial morphology and cristae. Finally, using RT-qPCR and WB, an increase in the expression of mitochondrial fusion proteins Mfn1, Mfn2, and Opa1 after PL1-2 treatment was observed, coupled with a decline in the expression and phosphorylation of mitochondrial cleavage proteins Fis and Drp1. These findings collectively demonstrate that PL1-2 ameliorates renal fibrosis by reducing oxidative stress and restoring mitochondrial balance.

## Introduction

Renal fibrosis (RF), an affliction marked by the formation of scars within the renal parenchyma, stands as the ubiquitous culmination across nearly all chronic and advancing renal maladies [1]. An array of epidemiological investigations have unveiled a worldwide surge in the incidence of end-stage renal disease [2]. Despite the evaluation of various therapeutic strategies, encompassing gene therapy, protein and peptide-based therapeutics, as well as plant-derived bioactive compounds, none have achieved the elusive feat of complete reversal of renal fibrosis [3, 4]. Consequently, a multitude of patients confronted with advanced stages of the disease must resort to kidney transplantation as a means to prolong their existence.

The kidneys possess the crucial role of upholding electrolyte, water, and acid-base equilibrium while purging the body of harmful waste materials. Essentially, the glomerulus is a selective filter about 60 kDa in size. This extensive permeability necessitates the renal tubule to diligently reabsorb 99% of the filtrated minute molecules, a process that demands substantial energy. The proximal renal tubule cells heavily rely on mitochondria to sustain their cellular function and viability. Mitochondria, being highly dynamic organelles, adroitly adapt to an array of stressful conditions through continuous fusion and fission, ensuring the cell’s energy metabolism [5]. This biological phenomenon is referred to as mitochondrial dynamics. The regulation of mitochondrial dynamics is mediated by a multifaceted interplay between fusion proteins [Mitofusin1/ 2 (Mfn1/ Mfn2) and Optic atrophy protein 1 (Opa1)] and fission proteins [Dynamin-related protein 1 (Drp1) and mitochondrial fission 1 (Fis1)] [6]. Fusion serves to bind the mitochondrial components to the vigorous mitochondria via complementation, augmenting oxidative phosphorylation while diminishing the presence of damaged mitochondria. On the other hand, fission is essential for the generation of fresh mitochondria. Nevertheless, an excess of mitochondrial fission leads to the generation of copious amounts of reactive oxygen species (ROS), thereby engendering an acute surge in intracellular ROS, consequently instigating drp1-mediated mitochondrial fission [7]. Profound abnormalities in mitochondrial dynamics have been disclosed in various models of experimentally-induced renal fibrosis [8, 9]. Studies have demonstrated notable reductions in the levels of Mfn1/2 and Opa1 in various pathological processes [10]. Conversely, pathological conditions elicit an elevation in the expression levels of fission proteins Drp1 and Fis1 [11, 12]. Therefore, targeting the modulation of mitochondrial kinetic homeostasis and reduction of reactive oxygen species production holds promise as potential therapeutic approaches in the development of new strategies against renal fibrosis.

*Panax notoginseng* (Burk.) roots F. H. Chen, commonly referred to as Sanqi or Sanchi in China, are a variant of ginseng herb hailing from the esteemed Araliaceae family. Researchers have utilized various analytical techniques to isolate a range of chemical constituents from Panax notoginseng roots. These constituents include saponins, flavonoids, polysaccharides, cyclic peptides, polyacetylene, sterols, volatile oils, and amino acids [13]. One of the most distinctive bioactive components found in Panax notoginseng is Panax notoginseng saponins (PNSs), which make up approximately 15% of the total content [14]. Within the PNSs, notoginsenoside R1, ginsenoside Rb1, Rg1, Rd, and Re have been extensively investigated in current pharmaceutical experiments, collectively accounting for 90% of the total PNSs. Their respective percentages in PNSs are 7%-10%, 30%-36%, 20%-40%, 5%-8.4%, and 3.9%-6% [15]. For clarity, the structures of the major saponins in Panax notoginseng are depicted in S1 Fig. Previous studies have revealed that comprehensive saponins derived from Panax notoginseng have a remarkable ability to modulate mitochondrial energy metabolism, oxidative stress, biosynthesis, and apoptosis [14]. Furthermore, these saponins have shown significant potential in impeding the progression of renal fibrosis in various animal models of renal failure [16–18]. *Leech* (Whitmania pigra whitman; Chinese Pinyin: Shui Zhi), renowned for their therapeutic properties in enhancing blood circulation and alleviating blood stasis, have been utilized in medical applications [19]. Moreover, leech have demonstrated the ability to enhance glomerular filtration rate, restore renal function, and address renal fibrosis [20]. The primary chemical constituent within leech, hirudin, has been found to enhance mitochondrial membrane potential and reduce the production of ROS [21].

In our initial investigation, we discovered that the combination of these two treatments yielded more efficacious prevention against kidney fibrosis compared to monotherapy with either substance [22]. However, the precise mechanism underlying this phenomenon remains unclear. Therefore, in the present study, we aimed to adjust the ratio of these two drugs to determine an optimal synergy while exploring the potential relationship between their mode of action, oxidative stress, and mitochondrial dynamics. This exploration will undoubtedly offer novel perspectives for the treatment of renal fibrosis.

## Material and methods

### Animals and drugs

Forty male Sprague-Dawley (SD) rats, aged 4 weeks and weighing 200±20 g, were subjected to a period of acclimatization feeding for one week. These rats were then randomly assigned to one of five distinct groups: control, RF, Panax notoginseng: leech 1:1 (PL1-1), Panax notoginseng: leech 2:1 (PL2-1), and Panax notoginseng: leech 1:2 (PL1-2), with each group consisting of eight rats. The control group was administered a daily dose of 10 mL/kg of distilled water, while the remaining groups were administered the same amount of adenine suspension (250 mg/kg/day) via gavage, sourced from Solaribio company in China. The induction of RF was carried out over a duration of 4 weeks, meticulously ensuring a standardized modeling period for all subjects.

Panax notoginseng and leech powder were obtained from Taiji Group Sichuan Mianyang Pharmaceutical Co Ltd (2001001, 2001003, Sichuan, China) and authenticated by Prof. Huang Xuekuan of Chongqing Medical University, both satisfying the requisites outlined in the 2020 edition of the Pharmacopoeia of the People’s Republic of China. The total amount of PL in the treatment was 1.2 g/kg/d, calculated based on the dose specifications prescribed in the aforementioned Pharmacopoeia and the rat-human conversion ratio combination. Dissolve Panax notoginseng powder and leech powder in water according to the grouping ratio (1:1, 2:1, and 1:2). In the treatment group, the gavage volume was 0.5 ml / 100 g, while the control group got an equivalent amount of distilled water. At the end of the experiment, the rats were sedated intraperitoneally with 3% pentobarbital, asphyxiated with high amounts of carbon dioxide, and executed. All experiments strictly adhered to the ethical institutional standards. The study protocol had been authorized by the Ethics Committee of Chongqing Medical University, China (Ethics Committee number: 2022165).

### Detection of metabolic and biochemical indicators

The rats’ weight was measured on a weekly basis. Furthermore, urine samples were taken in metabolic cages for 24 hours, and urine production, food intake, and water intake were all monitored. Urinary protein concentration was assessed according to the manufacturer’s instructions, and urinary proteins were quantified after 24 hours using urine volume and urinary protein concentration. The blood was centrifuged at 3000 rpm for 10 minutes, and the supernatants were collected for subsequent use. The matching detection kits from Nanjing Jiancheng Bioengineering Institute (Nanjing, China) were used to assess blood urea nitrogen (BUN) and serum creatinine (Scr), glutathione (GSH), superoxide dismutase (SOD), and malondialdehyde (MDA).

### Hematoxylin-eosin (HE) staining

Deparaffinized paraffin sections were stained for 5 minutes with a HE staining solution (G1003, Servicebio, China), rinsed with tap water, differentiated with a differentiation solution, rinsed with tap water, returned to blue with a return to blue solution, and rinsed with running water. The sections were then dehydrated in 85% and 95% gradient alcohol for 5 minutes each before being stained with an eosin staining solution for 5 minutes. The slices were finally dehydrated, sealed, and viewed under a microscope.

### Masson staining

Paraffin sections were deparaffinized and stained using the Masson staining set (G1006, Servicebio, China). The sections were immersed in Masson A liquid overnight, washed, and submersed for 1 min in a mixture of Masson B and Masson C liquids, in equal proportions, washed with water, and then differentiated with a solution of 1% hydrochloric acid and alcohol. Subsequently, they were immersed in Masson D liquid for 6 min, followed by a 1-minute dip in Masson E liquid, and a brief 30 second dip in Masson F liquid. The sections were rinsed and differentiated with 1% glacial acetic acid and finally dehydrated in two successive tanks of anhydrous ethanol. The sections were sealed with transparent film, and subjected to microscopic examination, and the images were captured and analyzed.

### Immunohistochemistry (IHC)

Paraffin sections were dewaxed using a gradient ethanol treatment. Antigen retrieval was achieved by steam heating in a citrate-based buffer, followed by blocking with 3% H_2_O_2_. Following a PBS rinse, sections requiring repair were immersed in a citrate buffer (final concentration of 0.01 mol/L) in an autoclave, boiled for 2 minutes, and allowed to cool naturally at room temperature. Subsequently, 100 μL of non-immune normal goat serum was added dropwise after rinsing with PBS, and the sections were incubated at room temperature for 60 min.

Primary antibodies against Col-IV, α-SMA, and FN (AiFang biological, China) were added dropwise at dilutions of 1:100, 1:500, and 1:100, respectively, following the removal of the blocking solution with a pipette gun. The samples were then incubated overnight at 4°C. After a PBST wash, an immunohistochemistry secondary antibody was applied and allowed to react for 30 minutes at room temperature. Following a second rinse, sections underwent 3,3’-diaminobenzidine color development, hematoxylin re-staining, alcohol differentiation using 1% hydrochloric acid for color restoration, gradient alcohol dehydration, and xylene treatment for transparency. A drop of neutral gum was placed at the center of each paraffin section, covered with a coverslip, sealed, and then subjected to microscopic examination.

### Ultra-performance Liquid Chromatography and Tandem Mass Spectrometry (UPLC-MS/MS) analysis

For the acquisition conditions of mass spectrometry chromatography tandem mass spectrometry and ultra-high performance liquid chromatography, the main liquid phase conditions were as follows. The chromatographic column used was the Agilent SB-C18 HPLC column with a particle size of 1.8 μm, measuring 2.1 mm × 100 mm. The mobile phases consisted of phase A, which was ultrapure water containing 0.1% formic acid, and phase B, which was acetonitrile containing 0.1% formic acid. The elution gradient started at 0.00 min with a proportion of phase B at 5%. Over the next 9 minutes, the proportion of phase B linearly increased to 95% and was maintained for 1 minute. From 10.00 to 11.10 minutes, the proportion of phase B decreased to 5% and was maintained for 14 minutes. The flow rate was set at 0.35 mL/min, the column temperature was maintained at 40°C, and the injection volume was 2 μL.

Moving on to the mass spectrometry conditions, the electrospray ionization (ESI) source was set at 500°C, and the ion spray voltage (IS) was adjusted to 5500 V for positive ion mode and -4500 V for negative ion mode. The ionization source gases (GS) I and II, as well as the curtain gas (CUR), were set to parameters of 50, 60, and 25 psi, respectively. Collision-induced ionization was set to a high level. The multiple reaction monitoring (MRM) mode was chosen for QQQ scanning, and the collision gas (nitrogen) was set to a medium level. Further optimization was performed for individual MRM ion pairs, considering the de-clustering voltage and collision energy. Finally, each period was examined based on specific sets of MRM ion pairs corresponding to the metabolites eluted during that period.

### Enzyme-linked Immunosorbent Assay (ELISA)

Rat serum ROS levels were quantified using a kit (Jiangsu Meinian Industrial Co., Ltd, Yanchen, China). Samples and standards were prepared and added according to the manufacturer’s instructions. They were incubated at 37°C for 30 minutes. The plate was washed five times, followed by the addition of the enzyme labeling reagent and incubation at 37°C for 30 minutes. After another five washes, color development solutions A and B were added, and the color was allowed to develop for 10 minutes. Finally, the termination solution was added, and the optic density was read within 15 minutes. Calculations were performed according to manufacturer’s guidelines.

### Transmission Electron Microscopy (TEM)

Renal cortical tissue was collected and cut into 1m^3^ cubes. The tissue cubes were then immersed in electron microscope fixative and washed three times with 0.1M phosphate buffer for a total of 15 minutes. The samples were then embedded with acetone and the 812-embedding compound after fixation in osmium tetroxide and drying in an ethanol solution. After 48 hours of polymerization in the oven, the resin block was sliced into 70 nm sections using an ultrathin slicer, and these sections were transferred onto a 150-mesh copper grid. The copper grid was stained with 2% uranyl acetate in a saturated alcoholic solution protected from light and 2.6% lead citrate solution protected from carbon dioxide. After drying, the sections were examined using a transmission electron microscope, and images were captured and analyzed.

### Real-time Quantitative Polymerase Chain Reaction (RT-qPCR) analysis

Real-time quantitative RT-qPCR was used to measure the levels of mRNA expression of alpha-smooth muscle actin (α-SMA), fibronectin (FN), collagen IV (Col Ⅳ), Mfn1/2, Drp1, Opa1, and Fis 1 in the renal tissue. Total RNA was isolated from renal tissue using the Trizol reagent, reverse transcribed into cDNA, and then mixed with 2× SYBR Green Pro Taq HS Premix according to the manufacturer’s protocols. The thermal cycle protocol included an initial 95° C cycle for 30 s, followed by 40 cycles of 95°C for 5 s, and a cycle of 60°C for 30 seconds (Accurate Biology, China). Ct values were standardized by using β-actin as a housekeeping gene. The relative quantification method (2^−ΔΔCt^) was used to calculate fold changes in mRNA expression. Table 1 lists the primer sequences used in RT-qPCR.

**Table 1:** Primer sequences used for RT-qPCR.

Primers	Forward	Reverse
β-actin	ATACAGGGCTTTCGCTTCAGT	CCCAGGTCACCTCGACGTT
α-SMA	GGGAGTGATGGTTGGAATGGG	CCGTTAGCAAGGTCGGAT
Col-IV	ACACTCCGACACCCATACAG	AGCACCGTCTTTTCCAGGTT
Mfn1	ATCAAGGAGGTCACGGAGGA	ACCAAAACAGACAGGCGACA
Mfn2	GGACTTTCACCCATCCCCAG	GATTCCGCACAGACACAGGA
Opa1	GGTTGCTTGGGAGACCCTAC	GTTCCATTTGTGCCGCTTGA
Drp1	GCTGCCTCAGATTGTCGTAG	ACTCCATTTTCTTCTCCTGTTGT
Fis1	CGCCTGCCGTTACTTCTTC	GTTCATCCCTTACCACGCAAC
FN	AGACCTGTGCCTGCCATTAC	GTTCGTGTCCCTTACTCCCTG

### Western Blot (WB)

We used RIPA lysis buffer to extract total proteins from renal tissue. Proteins were separated on a 10% sodium dodecyl-sulfate polyacrylamide gel electrophoresis gel and then transferred to methanol-activated polyvinylidene fluoride membranes (Merck Microporous, Germany). After blocking with 5% skimmed milk for 60 min, the membranes were incubated overnight at 4°C with primary antibodies against GAPDH (1:10000, SAB, China), α-SMA (1:25000, Abcam, UK); Col Ⅳ (1:800, AiFang, China), FN (1:500, AiFang, China), Mfn1 (1:1000, AiFang, China); Mfn2 (1:1000, AiFang, China); Drp1 (1:1000, AiFang, China); p-Drp1 (1:1000, AiFang, China). The next day, after three washes with Tris-buffered saline with 0.1% Tween-20 (TBST) buffer, the protein samples were incubated with horseradish peroxidase-labeled Goat Anti-Rabbit IgG secondary antibody (1: 5000, Epizyme Biotech, China) for 1 hour at 37°C. Blots were visualized using an electrochemiluminescence reagent (Abbkine, China), and the intensity of the bands was evaluated using Image J.

### Statistical analysis

Data is presented as mean ± standard error of mean (SEM). Statistical analysis was performed using GraphPad Prism 8.4.3 (GraphPad Software, La Jolla, CA, USA). Normality was assessed with Shapiro-Wilk tests. Statistical significance was assessed through one-way ANOVA and post-hoc Tukey’s multiple comparison tests. Results were considered statistically significant for *p*-levels < 0.05.

## Results

### PL improved renal function and increased body weight

**Fig 1:**
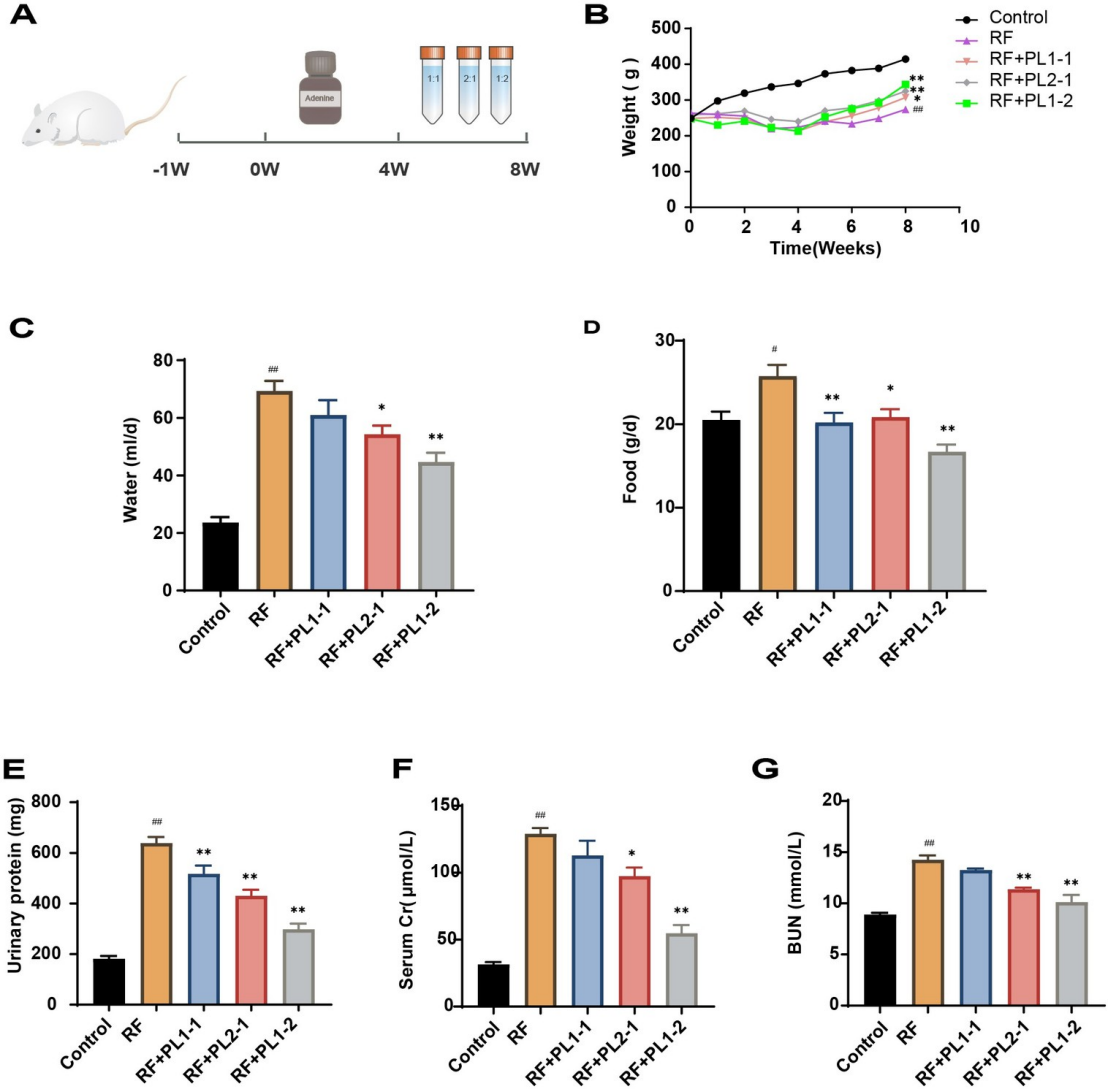
PL improved renal function and increased body weight. (**A**) Animal experiment timeline. (**B**) Body weight evolution. (**C-D**) The 24-hour food and water intake during the last week of the pharmacologic intervention. (**E-G**) BUN and Scr content following pharmacological intervention. (n≥3). Data are expressed as mean ± SEM; ^#^*p* < 0.05, ^##^*p* < 0.01 versus the control group; *p < 0.05, **p < 0.01, versus the RF group.

We conducted a study to investigate the antifibrotic effects of PL in vivo using an adenine-induced RF rat model (Fig 1A). During the initial 4 weeks, the RF rats modeled by adenine exhibited a distinctively lower body weight compared to the control group (*p* < 0.01), and their weight was continuously decreasing. However, upon treatment with different ratios of PL from weeks 5–8, there was a significant increase in body weight observed in the treatment group compared to the RF group (*p* < 0.05) (Fig 1B). Furthermore, we observed that the RF group consumed a significantly higher amount of water over 24 hours compared to the normal control group, whereas the PL-treated group showed a decrease in water intake (Fig 1C). Similarly, the RF group exhibited notably higher 24-hour food intake compared to the normal group (*p* < 0.05), which was significantly reduced in the PL-treated group (*p* < 0.05) (Fig 1D). Among the PL-treated groups, namely PL1-1, PL2-1, and PL1-2, there was a lower food and water intake overall. Notably, the PL1-2 group demonstrated the most substantial difference compared to the RF group.

To assess renal function, we measured the levels of 24-hour urinary protein, Scr, and BUN. The RF group displayed significantly higher levels of 24-hour urine protein quantification, as well as Scr and BUN serum levels, compared to the normal control group (*p* < 0.01). However, the PL-treated groups showed a reduction in these parameters, with the most substantial effect observed in the PL1-2 group (Fig 1E–1G). These findings indicate that PL effectively reduces metabolic abnormalities such as polydipsia, polyphagia, polyuria, and weight loss in RF rats, while also improving renal function.

### PL alleviated renal pathological injury

**Fig 2:**
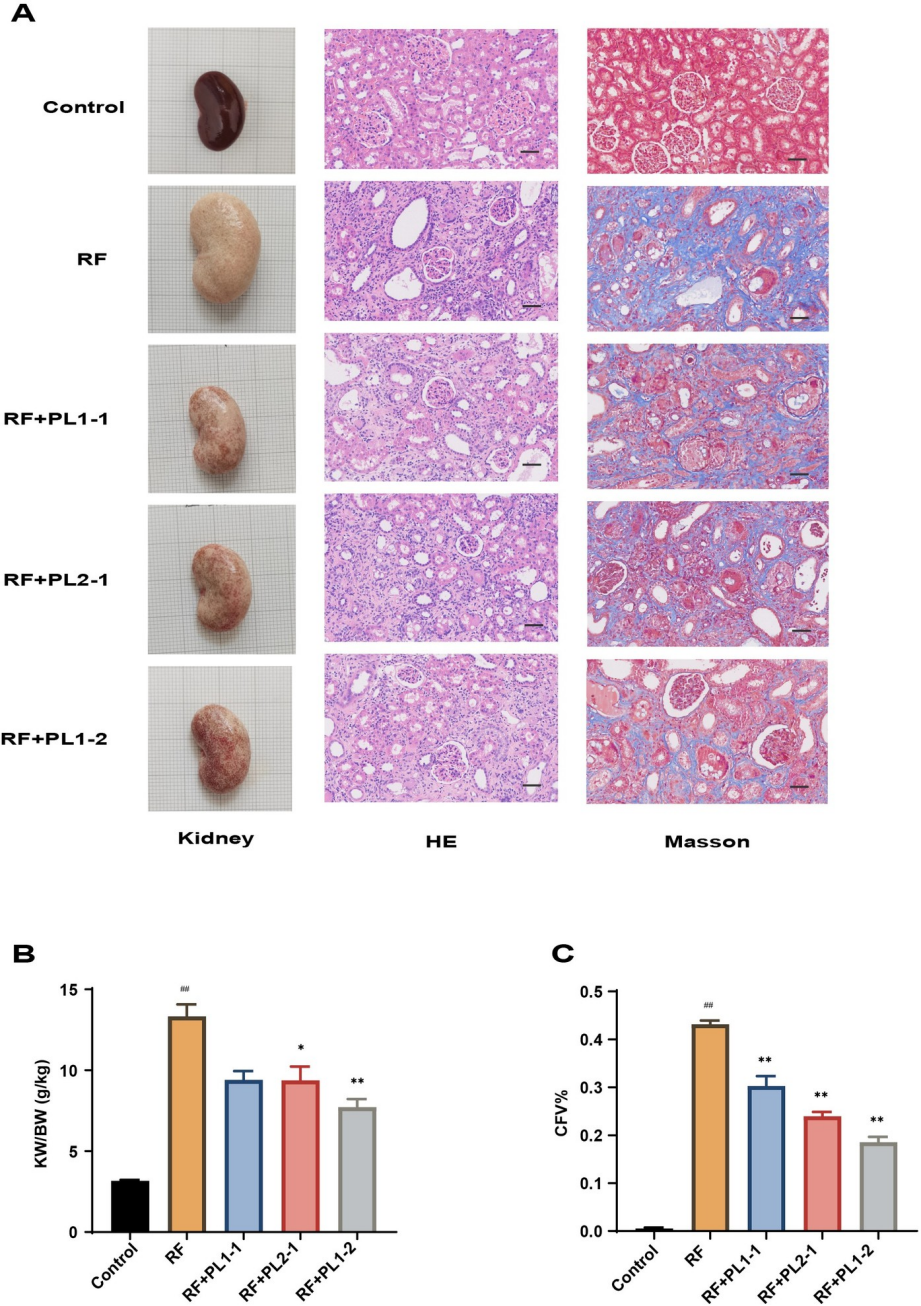
PL alleviated renal pathological injury. (**A**) Renal pathological changes evaluated by visual analysis, as well as hematoxylin-eosin and Masson stainings (scale bar = 20 μm). (**B**) The extent of renal fibrosis (n≥3) assessed using ImageJ software. (**C**) Left kidney and body weight after pharmacological intervention (n≥3). Data are expressed as mean ± SEM; ^#^*p* < 0.05 and ^##^*p* < 0.01 versus the control group; *p < 0.05, **p < 0.01 versus the RF group.

Pathologic features of the kidney with RF include parenchymal cell injury, immune cell infiltration, ECM deposition, and tubular atrophy [23, 24]. To analyze these pathological changes, we utilized HE and Masson staining techniques (Fig 2A–2C). In the normal group, the glomeruli and tubules exhibited a tightly packed, normal morphology with minimal inflammatory cell infiltration in the renal interstitium. Renal autopsy results revealed significantly enlarged kidneys with a white surface and kidney weight-to-body weight coefficients of approximately 13.34 in the RF group. After treatment with PL, restoration of blood supply was observed to varying degrees in the treated groups. The kidney weight-to-body weight coefficients were reduced to 9.41 in the PL1-1 group, 9.38 in the PL2-1 group, and 7.71 in the PL1-2 group, indicating a reduction in renal edema. Analysis of HE results demonstrated the presence of massive inflammatory cell infiltration, irregular dilatation, and atrophy of renal tubules in the RF group. However, PL treatment effectively restored renal tubular morphology and reduced renal interstitial inflammation. It is worth noting that the PL1-2 group exhibited less reduction in inflammatory cell infiltration compared to the PL1-1 and PL2-1 groups, which could potentially be attributed to the stronger anti-inflammatory effect of leech. Furthermore, Masson staining revealed significant collagen fiber deposition in the RF group, characterized by a collagen volume fraction (CFV) of approximately 0.43. However, after treatment, the CFV significantly decreased (p < 0.01) to 0.30 in the PL1-1 group, 0.24 in the PL2-1 group, and 0.19 in the PL1-2 group, respectively, indicating that PL effectively reduced collagen fiber deposition. These findings demonstrated that PL, especially PL1-2, was highly effective in improving the renal pathology in RF rats.

### PL improved renal fibrosis

**Fig 3:**
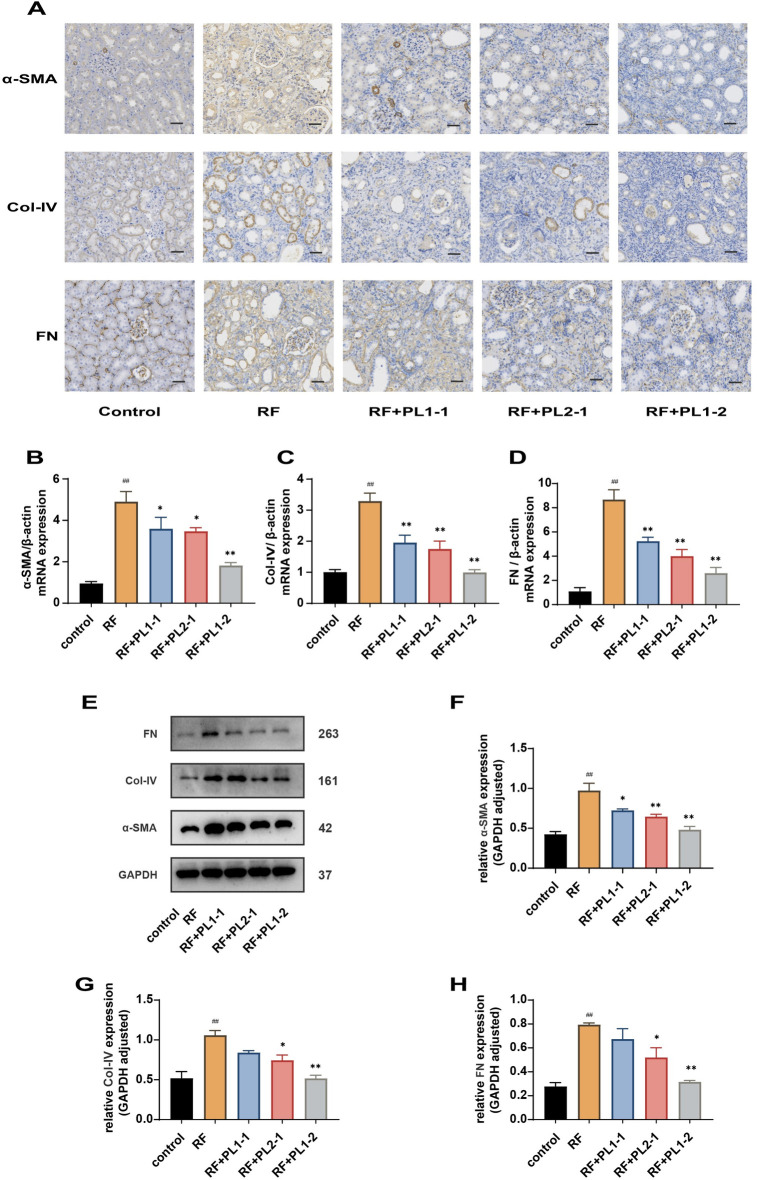
PL improved renal fibrosis. (**A**) Expression levels of FN, Col Ⅳ, α-SMA assessed using IHC. The scale bar corresponds to 20 μm. (**B-D**) mRNA expression levels of α-SMA, Col Ⅳ, and FN in the renal tissue evaluated using RT-qPCR (n≥3). (**E-H**) Protein levels of α-SMA, Col Ⅳ, and FN in the renal tissue assessed using WB. Image J was used for the visualization and quantification of protein bands (n≥3). Data are expressed as ±SEM; ^##^*p* < 0.01 versus the control group; *p < 0.05, **p < 0.01 versus the RF group.

Renal fibrosis is characterized by a massive accumulation of ECM. In this study, we aimed to evaluate the protective effect of PL against renal fibrosis by utilizing IHC staining, RT-qPCR, and WB. α-SMA, Col IV, and FN were identified as the major components of ECM. The positive IHC staining appeared as a brown color. The results demonstrated a marked accumulation of ECM in the renal tissue of the RF group compared to the control group (*p* < 0.01). However, treatment with PL effectively reduced the accumulation of ECM (Fig 3A). To further analyze the effects of different ratios of PL, we quantitatively evaluated α-SMA, Col IV, and FN through RT-qPCR and WB analysis (Fig 3B–3H). The mRNA and protein levels indicated that PL1-1, PL2-1, and PL1-2 prominently reduced the expression levels of α-SMA, Col IV, and FN compared to the RF group (*p* < 0.05), with the most pronounced effect observed in the PL1-2 group (*p* < 0.01). Therefore, based on these compelling results, it can be concluded that the PL1-2 group was the most effective in improving renal fibrosis.

### Analysis of rat serum by UPLC-MS/MS

**Fig 4:**
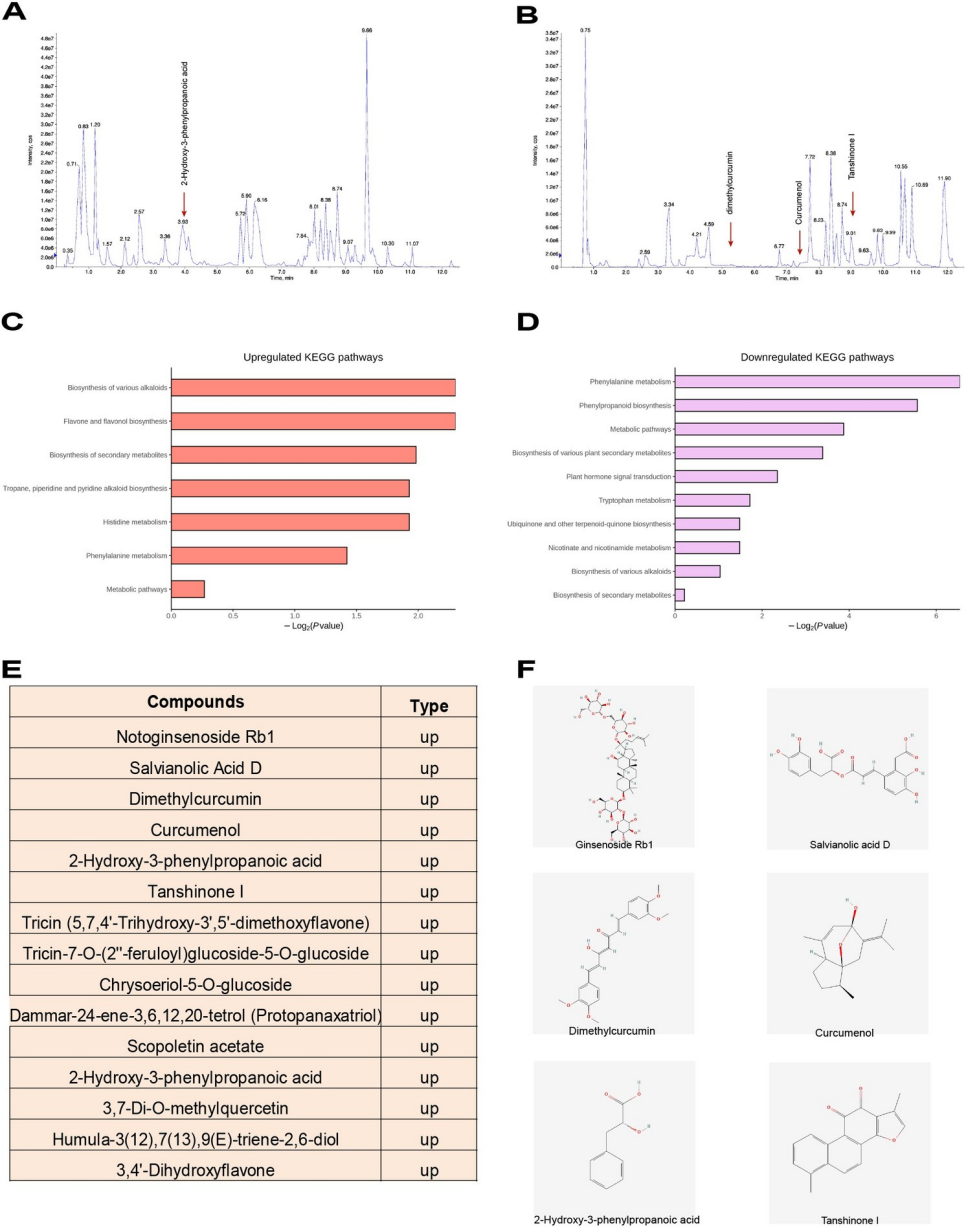
UPLC-MS/MS analysis of the serum of PL1-2-treated rats. Both the positive (**A**) and negative (**B**) ion modes of the chromatography are shown. (**C-D**) Changes in KEGG-related pathways. (**E**) The names of the components that are more differentiated. (**F**) Chemical formulations that have a strong correlation with the disease or mechanism under investigation. UPLC-MS/MS, ultra-performance liquid chromatography-tandem mass spectrometry.

Due to the superior therapeutic efficacy observed in previous studies, the PL1-2 group was selected as the representative for subsequent experiments, and its Ingredient was analyzed prior to initiation (S2 Fig). To better understand the mechanism of action of PL1-2, we conducted a UPLC-MS/MS analysis on the serum of PL1-2-treated rats to identify its differential chemical composition compared to that of the normal control group. The ion chromatograms in Fig 4A and 4B display the chemical composition of all the compounds. The KEGG pathway showed that PL mainly promoted the biosynthesis of various alkaloids, the biosynthesis of flavonoids and flavonols, and the metabolism of amino acids (Fig 4C). On the other hand, PL down-regulated the synthesis and metabolism of some amino acids (Fig 4D). We identified fifteen compounds with large differences between groups (Fig 4E), six of which had direct evidence of being strongly associated with restoration of mitochondrial function or alleviation of renal disease: Notoginsenoside Rb1, Salvianolic Acid D, Dimethylcurcumin, Curcumenol, 2-Hydroxy-3-phenylpropionic acid, and Tanshinone I. The remainder were flavonoids, terpenoids, or phenolic acids closely related to antioxidants (Fig 4F).

### PL1-2 reduced oxidative stress

**Fig 5:**
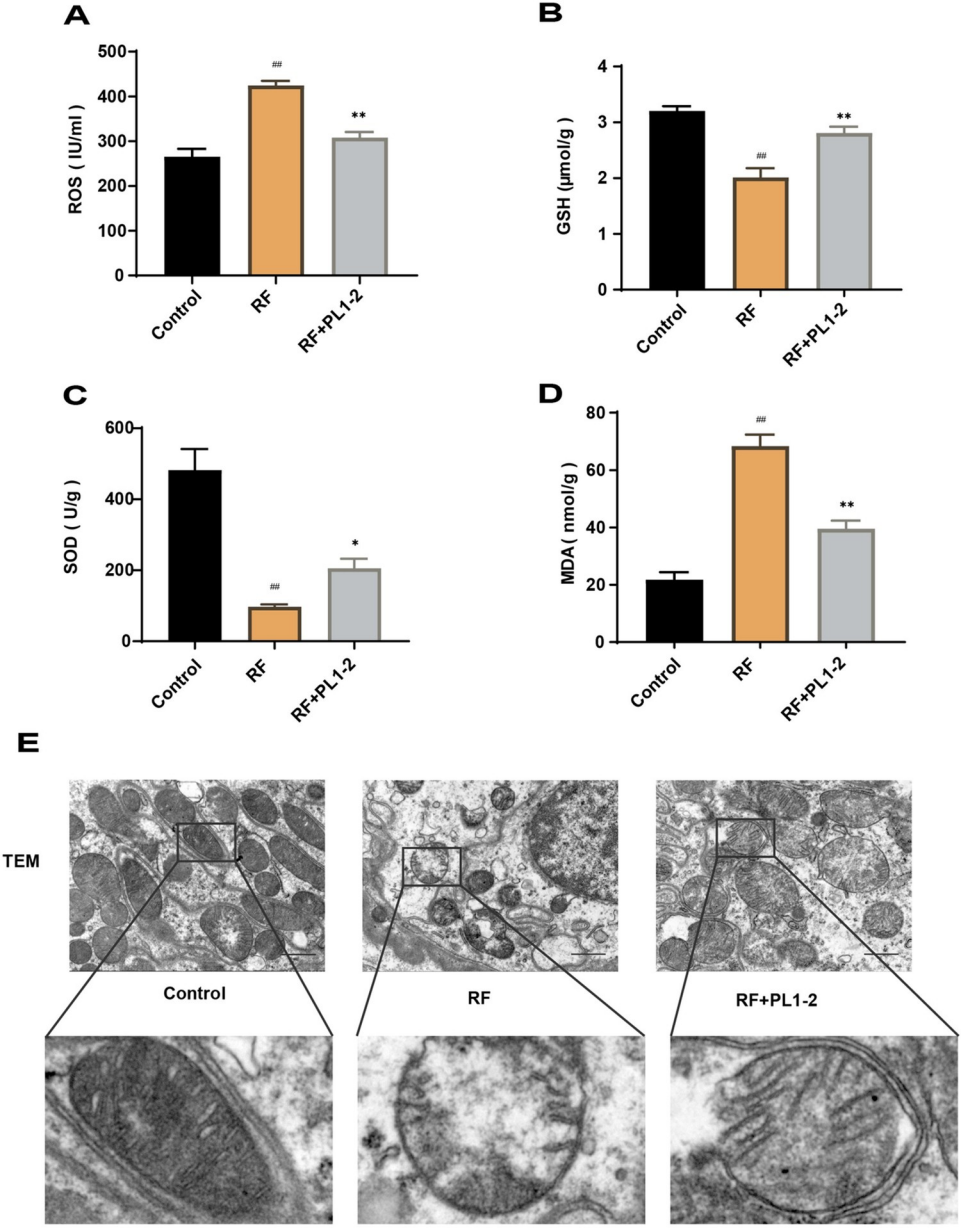
PL1-2 reduced oxidative stress. (**A**) The levels of reactive oxygen species in the renal tissue assessed using ELISA (n≥3). (**B-D**) Levels of glutathione, superoxide dismutase, and malondialdehyde following pharmacological intervention (n≥3). (**E**) Mitochondrial morphology of renal tissue following pharmacological intervention. The scale bar represents 10 μm. A lower mitochondrial microstructure was observed after a 5-fold enlargement. Data are expressed as mean± SEM; ^##^*p* < 0.01 versus the control group; *p < 0.05, **p < 0.01 versus the RF group.

Based on our study of serum levels of PL1-2 components, which suggested the presence of potent antioxidants, we hypothesized that PL1-2 could mitigate fibrosis by counteracting oxidative stress. To confirm this hypothesis, we examined ROS levels in renal tissues using ELISA (Fig 5A) and evaluated oxidative stress indicators, including GSH, SOD, and MDA, using biochemical kits (Fig 5B–5D). Remarkably, the renal tissue of rats in the RF group exhibited lower levels of SOD and GSH, and higher levels of MDA and ROS, compared to normal control rats (*p*<0.01). In contrast, the renal tissue of rats in the PL1-2 group displayed higher levels of SOD and GSH, and lower levels of MDA and ROS, compared to the RF group (*p*<0.05).

Considering that mitochondria are the primary source of ROS and excessive ROS can lead to mitochondrial damage, we utilized transmission electron microscopy to examine the morphology and structure of renal tissue mitochondria (Fig 5E). Astonishingly, kidney mitochondria in the RF group exhibited swelling, loss of cristae, and fragmentation when compared to the normal group. In contrast, renal mitochondria in the PL1-2 group demonstrated intact morphology, partial recovery of cristae, and reduced swelling compared to the RF group. These compelling findings strongly suggest that PL1-2 effectively alleviated oxidative stress, which may be the primary mechanism underlying its ability to mitigate mitochondrial damage.

### PL1-2 restored mitochondrial dynamics

**Fig 6:**
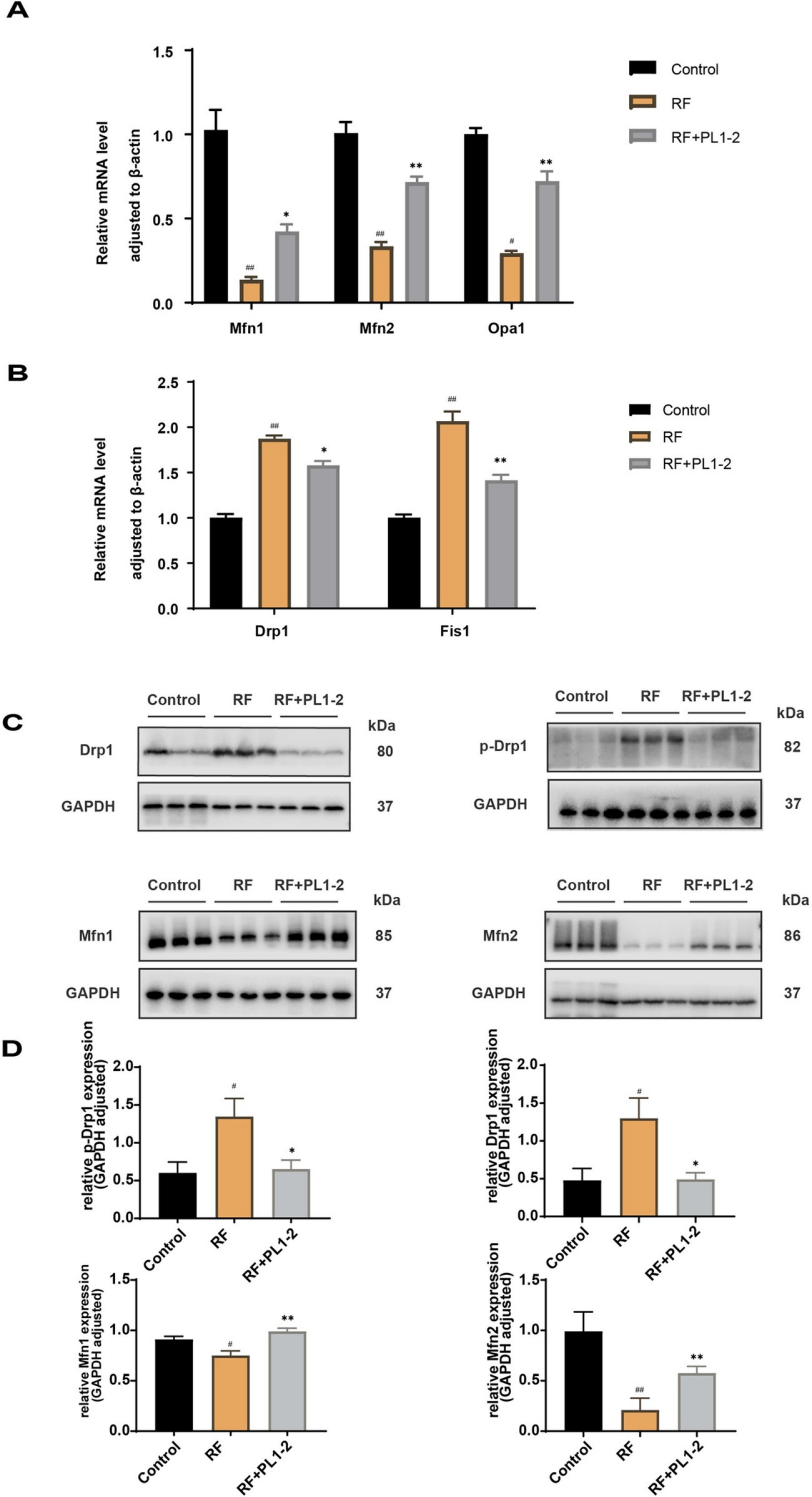
PL1-2 restored mitochondrial dynamics. (**A-B**) mRNA expression levels of Mfn1/2, Opa1, Drp1, and Fis1 in the renal tissue assessed using real-time quantitative polymerase chain reaction (n≥3). (**C**) Protein levels of Mfn1, Mfn2, Drp1, and p-Drp1 in the renal tissue assessed using WB (n≥3). (**D**) Bands were visualized and quantified using Image J. ^#^*p* < 0.05, ^##^*p* < 0.01 versus the control group; *p < 0.05, **p < 0.01 versus the RF group.

Disturbed mitochondrial dynamics predispose to renal fibrosis. In order to evaluate renal mitochondrial dynamics, we measured the expression of Mfn1, Mfn2, p-Drp1, and Drp1 using RT-qPCR and WB (Fig 6A–6D). Notably, in rats with RF, the expression levels of Mfn1 and Mfn2 were downregulated, while those of p-Drp1 and Drp1 were upregulated, indicating inhibition of mitochondrial fusion proteins and stimulation of cleaved proteins. Conversely, the PL1-2 group exhibited an upregulation in the expression levels of Mfn1 and Mfn2, and a downregulation in the expression levels of p-Drp1 and Drp1 compared to the RF group. Moreover, the RF group displayed an increase in Fis 1, a marker of mitochondrial fragmentation, when compared to the normal control, and this increase was mitigated by PL1-2. Importantly, these results were consistent with the findings observed through transmission electron microscopy. Therefore, PL1-2 plays a crucial role in partially mitigating renal fibrosis by restoring disturbed mitochondrial dynamics.

## Discussion

RF is a hallmark feature of various renal diseases and serves as the ultimate pathway for the progression of renal function decline [25]. By effectively improving RF, the progression of chronic renal failure (CRF) can be slowed down [26]. Traditional Chinese medicine has long utilized Panax ginseng and leeches for the treatment of fibrosis, with notable results [27–29]. Building on the effectiveness of these treatments, our study aims to explore the effects of different ratios of Panax notoginseng and leeches in alleviating RF and investigating the underlying mechanisms. In adenine-induced RF rats, we observed metabolic disturbances and renal dysfunction, characterized by increased tissue inflammation and collagen fiber accumulation, as indicated by HE and Masson staining. After four weeks of treatment with PL, we observed partial recovery of metabolism and renal function, with the most significant improvement observed in the PL1-2 group. Furthermore, myofibroblasts play a critical role in the production of FN and Col Ⅳ, which are key components of the ECM. Reducing the deposition of these fibrotic components is considered an important anti-fibrotic strategy [30]. The expression of α-SMA is also involved in regulating fibroblast-to-myofibroblast transformation. Analysis of mRNA and protein levels in kidney tissues from RF rats showed that PL1-1, PL2-1, and PL1-2 all significantly reduced the levels of collagen IV, fibronectin, and α-SMA. Compared to the PL1-1 and PL2-1 groups, the PL1-2 group exhibited superior therapeutic effectiveness. These findings indicate that PL therapy holds potential for protecting renal function and slowing down the progression of RF, with the PL1-2 ratio demonstrating the most potent antifibrotic effect.

PL1-2 group showed superior renal protection in these results, making it the ideal choice for mechanistic studies. Understanding the therapeutic actions of herbal medicines requires the extraction of their active ingredients. It is important to note that not all components of Chinese medicines are medicinal. However, in the case of oral herbal remedies, therapeutic compounds are more likely to be found in the blood of patients and animals who have consumed them [31]. Previous research has also shown that Ginsenoside Rb1 and Salvianolic Acid D not only protect against renal fibrosis but are also linked to the restoration of mitochondrial function [32–34]. Additionally, Dimethylcurcumin and curcumenol have demonstrated efficacy in treating renal disorders [35, 36]. Furthermore, chemicals such as 2-hydroxy-3-phenylpropanoic acid and tanshinone I have been proven to be effective antioxidants [37, 38]. According to the KEGG pathway, PL1-2 enhances the biosynthesis of flavonoids and flavonols, the biosynthesis of different alkaloids, and the metabolism of amino acids upon entry into the blood. These findings align with the results of our analysis of the key components of the medication. It is worth noting that alkaloids are derived from amino acids. Given that Panax notoginseng and leech are rich in amino acids, we hypothesize that the alkaloid production route is significantly altered in the presence of PL1-2.

Oxidative stress refers to the imbalance between oxidation and antioxidants. ROS are oxygen-containing free radicals and peroxides associated with oxygen metabolism in organisms [39]. Under normal conditions, both endogenous and exogenous antioxidants interact with these oxidants to counteract oxidative damage to cells. Key components of the antioxidant defense mechanisms are SOD and GSH, while MDA serves as a marker of membrane lipid peroxidation [40]. In cases of organism damage, excessive ROS deplete the levels of SOD and GSH. Additionally, oxidative damage disrupts the transcription of mitochondrial DNA, affecting the normal function and oxidative phosphorylation of mitochondrial proteins [41], which further hampers the production of SOD and GSH. Consequently, increased ROS in RF rats leads to decreased SOD and GSH levels and increased MDA levels. The results of our study demonstrate that PL1-2 was effective in reducing ROS production, decreasing body MDA levels, and increasing SOD and GSH levels. This indicates that PL1-2 enhanced the antioxidant capacity and attenuated injury-induced oxidative stress in RF rats.

**Fig 7:**
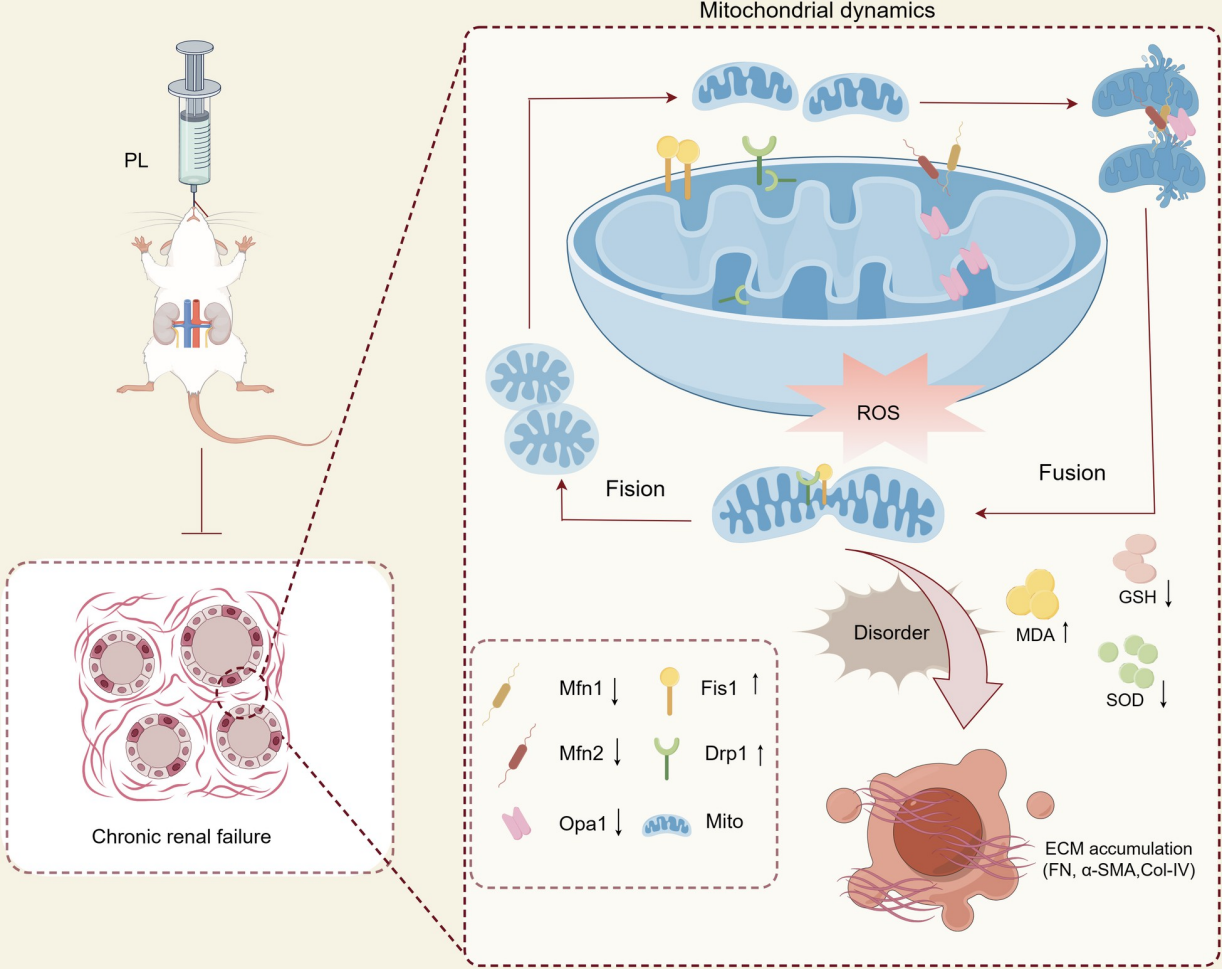
Diagram of the mechanisms of mitochondrial dynamics during chronic renal fibrosis.

Mitochondria being a major source of ROS and highly susceptible to ROS-mediated damage. Mitochondria are extremely dynamic organelles that regularly undergo cycles of fission and fusion to maintain their structure, function, heredity, and quality control [42]. Mfn1/2, located in the outer mitochondrial membrane, facilitates the fusion of MOM tethers between adjacent mitochondria, while Opa1, localized in the inner membrane, is essential for endosomal fusion. Together, they promote the formation of functional mitochondria by fusing defective ones, reducing cellular stress [6]. When externally stimulated, Drp1 moves from the cytoplasm to the outer membrane to initiate mitochondrial division [43]. In our study, we found that PL1-2 effectively increased the expression of Mfn1/2 and Opa1 while decreasing the expression and phosphorylation of Drp1 in the renal tissue of RF rats. We also observed a decrease in Fis1 expression, an indicator of mitochondrial fragmentation, which aligns with our TEM findings of reduced mitochondrial fragmentation. It is important to note that the aforementioned results pertain specifically to the effect of PL1-2 on mitochondrial dynamics in kidney tissue (Fig 7). However, further verification is required through subsequent cellular experiments to determine the accuracy of these findings at the cellular level.

In summary, the first part of this study demonstrated the superiority of PL1-2 in improving renal function, promoting tissue recovery, and inducing changes in the extracellular matrix to ameliorate RF. Subsequently, we conducted a study to understand the protective mechanism of PL1-2 in rats with adenine-induced RF. The results indicate that PL1-2 exerts a protective effect by mitigating oxidative stress and correcting disturbances in mitochondrial dynamics. This effect is achieved through the reduction of ROS accumulation, peroxidation product MDA, and the increases of SOD and GSH, thereby alleviating oxidative stress. Moreover, PL1-2 up-regulates mitochondrial fusion proteins Mfn1/2 and inhibits the activation of mitochondrial cleavage protein Drp1, effectively correcting the imbalance in mitochondrial dynamics in RF rats. These findings offer promising insights into the use of PL1-2 as a potential drug for addressing renal fibrosis and suggest new avenues for treating this challenging condition.

## Supporting information

S1 Fig The structures of the major saponinsin Panaxginseng. (TIF)

S2 Fig The drug’s component identification results. Typical sample (**A**) positive ion base peak plot and (**B**) negative ion base peak plot. The more similar the trends, the better the repeatability and the more reliable the results. (**C**) Classification of Chinese medicine identification results.
(TIF)

S1 Data (PDF)

S1 Raw images (PDF)
