# Supplementary figures and images for: Combined use of Panax notoginseng and leech provides new insights into renal fibrosis: Restoration of mitochondrial kinetic imbalance
Source: PLoS One. 2024 May 29;19(5):e0303906. doi: 10.1371/journal.pone.0303906 (PMC11135711; doi:10.1371/journal.pone.0303906)

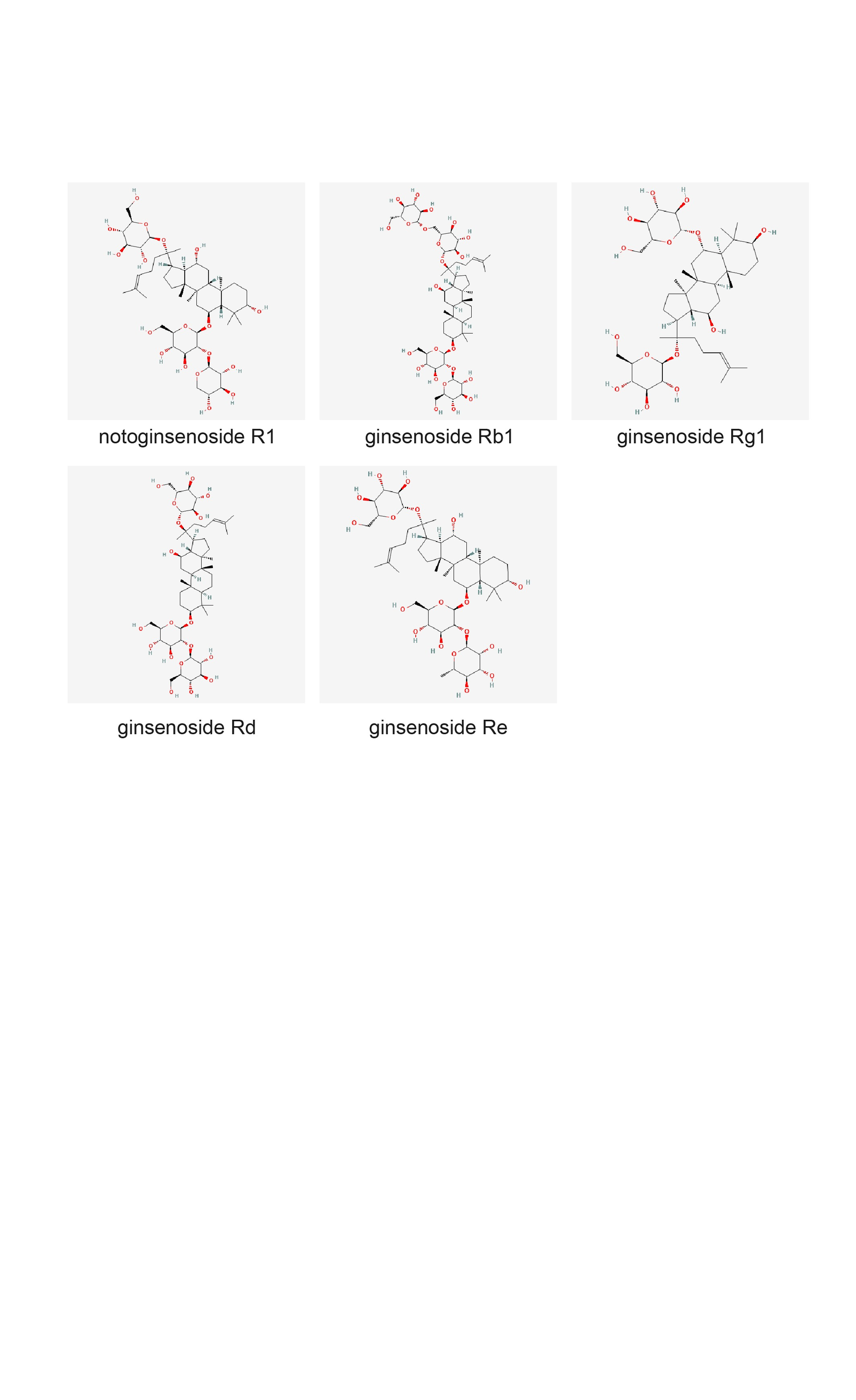

Supplement: S1 Fig — (TIF) [file pone.0303906.s001.tif]

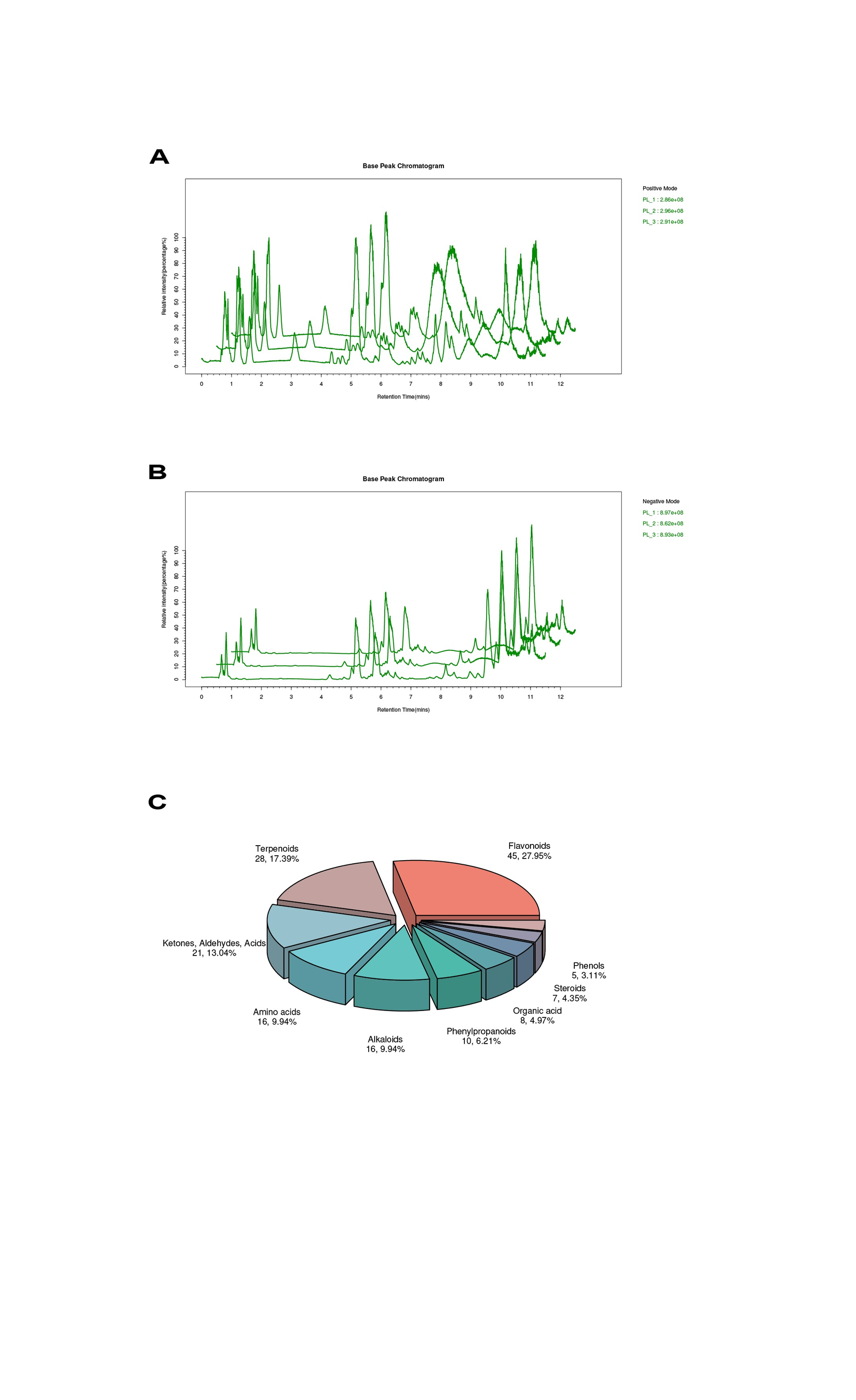

Supplement: S2 Fig — Typical sample (A) positive ion base peak plot and (B) negative ion base peak plot. The more similar the trends, the better the repeatability and the more reliable the results. (C) Classification of Chinese medicine identification results. (TIF) [file pone.0303906.s002.tif]
