# Supplementary material for: Combined use of Panax notoginseng and leech provides new insights into renal fibrosis: Restoration of mitochondrial kinetic imbalance
Source: PLoS One. 2024 May 29;19(5):e0303906. doi: 10.1371/journal.pone.0303906 (PMC11135711; doi:10.1371/journal.pone.0303906)
Supplement: S1 Data — (PDF) [file pone.0303906.s003.pdf]

Fig 1B

Time (Weeks)	control	RF	RF+PL1-1	RF+PL2-1	RF+PL1-2
0	248.975	260.95	249.0625	258.4875	250.9875
1	297.4625	255.45	251.725	261.675	236.15
2	319.25	252	247.6	269.375	244.8875
3	336.825	223.5625	223.8	246.0625	220.975
4	346.3	226.45	213.05	240.1375	212.2
5	373.5	231.4	239.125	270.225	255.95
6	382.5375	225.975	256.2	278.675	278.8625
7	388.6125	237.725	277.8875	298.3375	296.325
8	414.5	265.2	307.225	324.65	342.7

Fig 1C

control	RF	RF+PL1-1	RF+PL2-1	RF+PL1-2
29.2	82.1	67.7	43.4	57
15.5	71.6	49.8	52.6	46.4
26	80.4	62.9	68.6	50.7
24.6	67.6	53.8	43.7	52.8
24	75	71.5	54	44.5
28.7	63.1	79.9	58.9	37.8
25	54.8	68.6	53.4	29.8
16.4	60.4	33.8	60.2	38.7

Fig 1D

control	RF	RF+PL1-1	RF+PL2-1	RF+PL1-2
25	31.3	20.2	23.3	17.3
18.5	27.9	21.8	21.2	20.5
19.5	26.9	18.3	24.1	12.4
17	26.5	18.1	19.2	15.4
21.8	25.8	15.8	19	18.7
19	26.5	21	18.4	17.1
18.9	18.3	26.7	17.5	17.2
24.3	22.8	19.8	24	14.9

Fig 1E

control	RF	RF+PL1-1	RF+PL2-1	RF+PL1-2
136.9484937	563.2653061	667.755102	466.5087464	210.4373178
144.5286686	589.6598639	401.6326531	318.0515063	252.0894072
162.4878523	602.3517979	407.4052478	384.2614189	266.3945578
172.0699708	614.7521866	443.9455782	397.8620019	277.2594752
183.2653061	673.9067055	550.2332362	401.3605442	305.9280855
213.877551	770.8843537	553.2264334	469.5699708	323.4305151
217.866861	603.516035	557.1428571	488.1632653	337.3760933
220.2721088	690.3401361	560.4275996	520.1652089	413.9941691

Fig 1F

control	RF	RF+PL1-1	RF+PL2-1	RF+PL1-2
23.63082927	104.9588293	50.10770732	62.52682927	35.10126829
28.89170732	118.8440976	96.07570732	88.14126829	37.77482927
29.84039024	125.2261463	103.0614634	88.91746341	41.22458537
31.56526829	131.1769756	117.2917073	89.86614634	42.17326829
32.0626577	135.8341463	121.6901463	110.2197073	60.71570732
32.42770732	136.6965854	127.0372683	112.0308293	70.46126829
32.42770732	137.3002927	131.4357073	113.7557073	72.18614634
41.31082927	142.0437073	156.1877073	113.7557073	78.39570732

Fig 1G

control	RF	RF+PL1-1	RF+PL2-1	RF+PL1-2
8.510638298	12.9787234	13.82978723	10.85106383	5.744680851
8.510638298	13.19148936	12.76595745	11.06382979	8.723404255
8.723404255	13.40425532	12.76595745	11.27659574	10.21276596
8.723404255	13.82978723	12.9787234	11.27659574	10.63829787
8.723404255	13.82978723	12.9787234	11.27659574	10.63829787
9.14893617	15.31914894	13.40425532	11.4893617	11.27659574
9.14893617	15.31914894	13.40425532	11.70212766	11.70212766
9.787234043	16.17021277	13.82978723	12.12765957	11.91489362

Fig 2B

control	RF	RF+PL1-1	RF+PL2-1	RF+PL1-2
2.970036404	10.20713376	7.305381727	5.404849168	5.78057083
2.979176874	10.66446886	7.891035793	6.035770317	6.013589365
3.094322951	12.97069597	8.908684725	9.203992514	6.327262045
3.121142857	13.54568436	9.092252035	9.936947555	8.266827649
3.203522971	13.99611046	9.444559585	10.23784123	8.276457883
3.24007986	14.04511863	9.607130251	11.10385196	8.513055556
3.284251252	14.88806523	10.86540073	11.26540951	9.063404748
3.415944926	16.40151818	12.13065647	11.84308049	9.487563884

Fig 2C

control	RF	RF+PL1-1	RF+PL2-1	RF+PL1-2
0.01361746	0.45341861	0.3468442	0.21401201	0.21828008
0.00361426	0.44963375	0.23717412	0.22463273	0.15483175
0.0019093	0.41661298	0.25502825	0.23287968	0.17853078
0.00509266	0.44430503	0.33608389	0.24884515	0.17616551
0.00682694	0.41553161	0.28892034	0.27699876	0.22117475
0.00000000	0.40955007	0.35366765	0.24062268	0.16244824

Fig 3B

control	RF	RF+PL1-1	RF+PL2-1	RF+PL1-2
1.126013867	5.825912212	2.5666430	3.158667914	2.059976426
0.90443272	4.711428512	4.062084158	3.647210239	1.549684149
0.824902947	4.171178734	3.556225654	3.407091904	1.836557999

Fig 3C

control	RF	RF+PL1-1	RF+PL2-1	RF+PL1-2
0.890961283	3.107145033	2.339533110	1.471816849	1.145715517
1.164775524	3.797258650	2.013344096	1.520605096	0.832005996
0.963604749	2.974376025	1.515988831	2.258667914	1.005004780

Fig 3D

control	RF	RF+PL1-1	RF+PL2-1	RF+PL1-2
1.664645910	7.113575210	5.825912212	3.197091904	1.909575147
0.594623942	9.821594868	5.171178734	5.062084158	1.594921244
1.010266007	9.094244420	4.711428512	3.756225654	2.358346658

Fig 3F

control	RF	RF+PL1-1	RF+PL2-1	RF+PL1-2
0.47971754	0.997954467	0.762398709	0.696720788	0.549782526
0.435383433	0.800348264	0.697232093	0.64805697	0.489428323
0.359855259	1.120364246	0.712306184	0.592822396	0.404556459

Fig 3G

control	RF	RF+PL1-1	RF+PL2-1	RF+PL1-2
0.68610283	1.08554411	0.795381026	0.622027822	0.593129137
0.434317012	1.145526487	0.88344621	0.846568241	0.502479324

0.437655976	0.945320231	0.842262099	0.765188246	0.454487945

Fig 3H

control	RF	RF+PL1-1	RF+PL2-1	RF+PL1-2
0.333977093	0.809274136	0.845784364	0.589718835	0.293677695
0.276796379	0.807547024	0.616081323	0.612952843	0.336156464
0.221409461	0.767482007	0.559185988	0.356733012	0.317061248

Fig 5A

control	RF	RF+PL1-2
249.4606555	408.2865338	292.7853478
252.5619892	411.5758272	294.9938733
253.4078075	424.8269803	296.2156108
255.9452624	426.0487178	307.9160971
258.0128182	426.3306573	311.3463602
272.0158098	427.2704554	318.0659166
282.6355283	434.6948603	321.2612301
298.8940354	437.1383354	323.3287859

Fig 5B

control	RF	RF+PL1-2
2.9757	1.3533	2.3608
3.0056	1.4092	2.5298
3.042	1.8252	2.6195
3.1486	1.9604	2.6793
3.1499	2.2399	2.8587
3.2058	2.3075	3.0563
3.3631	2.314	3.0849
3.7258	2.6923	3.2929

Fig 5C

control	RF	RF+PL1-2
243.25	78.38	131.61
304.12	82.91	140.76
439.24	91.02	153.74
445.06	91.33	185.3
510.02	102.29	185.53
536.4	105.47	202.66
588.5	139.3	301.36
789.67	84.82	343.72

Fig 5D

control	RF	RF+PL1-2
11.4988	57.3325	22.2224
15.1487	57.8493	38.3401
15.6332	58.3984	38.5339
18.2818	61.3054	40.2781
24.4188	68.4437	41.021
29.1023	78.1983	41.6347
29.5868	78.812	44.0572
30.4589	86.3702	50.1619

Fig 6A	control	RF	RF+PL1-2
	0.957688587	0.195876499	0.498943089
	0.834654053	0.125248042	0.449904936
Mfn1	0.76422054	0.123190572	0.261021394

	1.144698378	0.134562319	0.425128704
	1.430076955	0.107887435	0.480503754
Mfn2	0.825137781	0.264180508	0.639146067
	1.127880122	0.35395833	0.695370952
	1.178039977	0.294983451	0.825099548
	0.96892954	0.409717488	0.674506388
	0.941365677	0.354781537	0.751106519
Opa1	1.025649115	0.254953358	0.705624602
	0.955695002	0.290853501	0.674098635
	0.959829177	0.329505931	0.543893087
	0.938295994	0.318161367	0.80603406
	1.132786503	0.279718995	0.881985714

Fig 6B	control	RF	RF+PL1-2
Drp1	1.093149637	1.856727161	1.636099016
	0.945510338	1.861415781	1.599125904
	1.052061408	1.807032415	1.674579345
	1.045222155	2.009117311	1.59573336
	0.879841427	1.831374727	1.393677896
Fis1	1.009455647	2.441180662	1.597079266
	1.000642756	2.12038164	1.34037245
	1.038224867	2.010580682	1.501839346
	0.876338182	1.953473157	1.374642348
	1.088104337	1.816372137	1.26418565

Fig 6D	control	RF	RF+PL1-2
p-Drp1	0.505091388	0.847187773	0.335790418
	0.222935623	0.779222786	0.650529784
	0.35490821	0.85089731	0.524830948
	0.678351954	1.789646367	0.968551064
	0.626371209	1.711922574	1.031624267
	1.228288236	2.095191926	0.400884851
Drp1	1.164383776	1.661721786	0.708633873
	0.517257374	2.081492662	0.574322656
	0.603349334	1.865989367	0.574486086
	0.102479324	0.921409461	0.116081323
	0.204487945	0.797547024	0.359185988
	0.276796379	0.467482007	0.612952843
Mfn1	0.971267769	0.741265754	0.950446156
	0.902687087	0.723728801	1.036212698
	0.922485611	0.634886165	1.047504372
	0.895212852	0.646733374	1.088138032
	0.991311787	0.884165588	0.881152586
	0.774577169	0.878907638	0.934388693
Mfn2	0.991105087	0.156764498	0.490561737
	0.969645198	0.117061248	0.576935586
	0.720639407	0.138784149	0.521181375
	1.234456399	0.199749836	0.620805405
	1.175640282	0.201382829	0.568828853
	0.850810623	0.442393492	0.676313831
