# Supplementary material for: Combined use of Panax notoginseng and leech provides new insights into renal fibrosis: Restoration of mitochondrial kinetic imbalance
Source: PLoS One. 2024 May 29;19(5):e0303906. doi: 10.1371/journal.pone.0303906 (PMC11135711; doi:10.1371/journal.pone.0303906)
Supplement: S1 Raw images — (PDF) [file pone.0303906.s004.pdf]

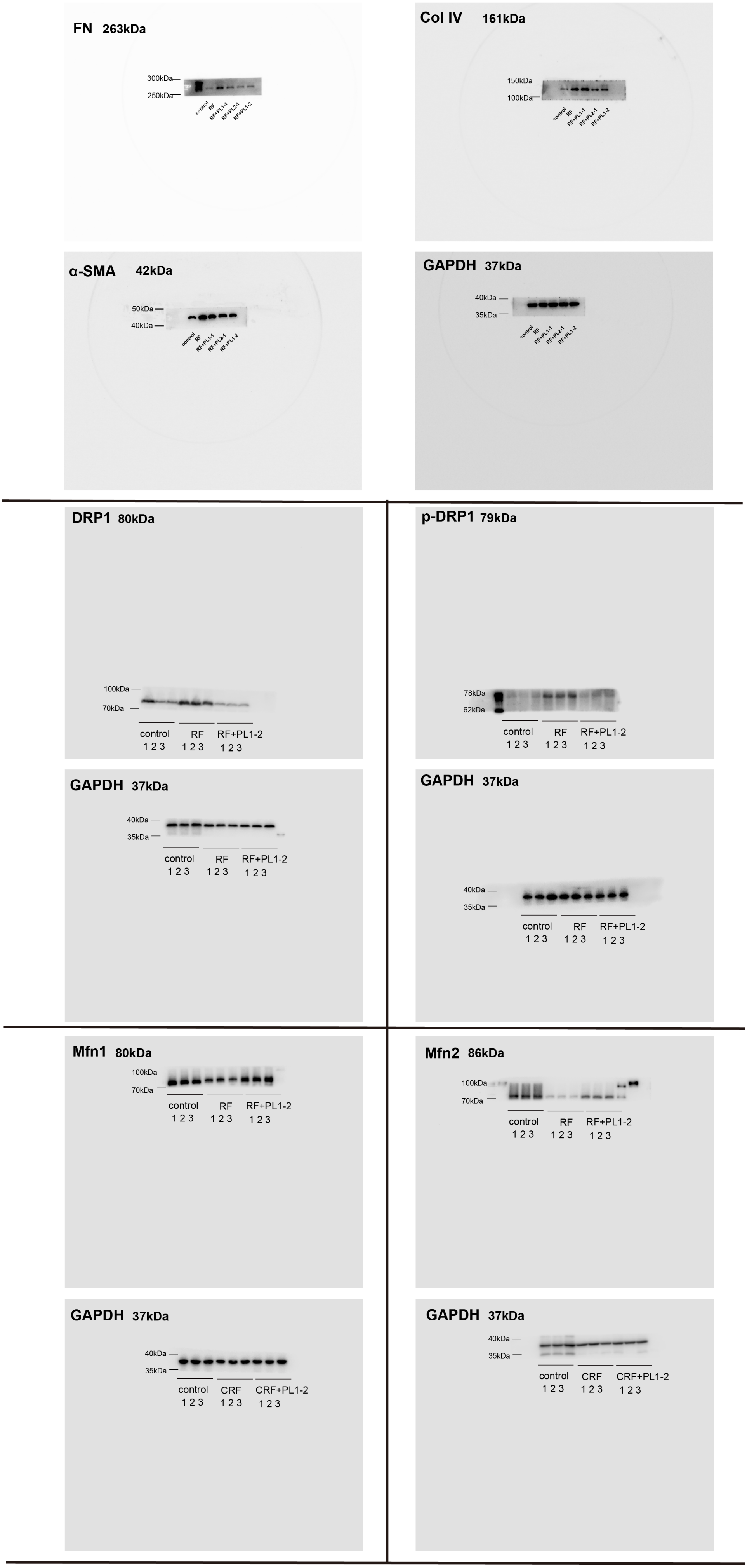
FN 263kDa
300kDa
250kDa
control
RF
RF+PL1-1
RF+PL2-1
RF+PL1-2
Col IV 161kDa
150kDa
100kDa
control
RF
RF+PL1-1
RF+PL2-1
RF+PL1-2
α-SMA 42kDa
50kDa
40kDa
control
RF
RF+PL1-1
RF+PL2-1
RF+PL1-2
GAPDH 37kDa
40kDa
35kDa
control
RF
RF+PL1-1
RF+PL2-1
RF+PL1-2
DRP1 80kDa
100kDa
70kDa
control RF RF+PL1-2
1 2 3 1 2 3 1 2 3
p-DRP1 79kDa
78kDa
62kDa
control RF RF+PL1-2
1 2 3 1 2 3 1 2 3
GAPDH 37kDa
40kDa
35kDa
control RF RF+PL1-2
1 2 3 1 2 3 1 2 3
GAPDH 37kDa
40kDa
35kDa
control RF RF+PL1-2
1 2 3 1 2 3 1 2 3
Mfn1 80kDa
100kDa
70kDa
control RF RF+PL1-2
1 2 3 1 2 3 1 2 3
Mfn2 86kDa
100kDa
70kDa
control RF RF+PL1-2
1 2 3 1 2 3 1 2 3
GAPDH 37kDa
40kDa
35kDa
control CRF CRF+PL1-2
1 2 3 1 2 3 1 2 3
GAPDH 37kDa
40kDa
35kDa
control CRF CRF+PL1-2
1 2 3 1 2 3 1 2 3
